# ^27^Al NMR Study of the pH Dependent Hydrolysis Products of Al_2_(SO_4_)_3_ in Different Physiological Media

**DOI:** 10.3390/molecules23040808

**Published:** 2018-04-02

**Authors:** Svend Berger, Jürgen Nolde, Timucin Yüksel, Wolfgang Tremel, Mihail Mondeshki

**Affiliations:** 1 GRACE Europe Holding GmbH, In der Hollerhecke 1, 67547 Worms, Germany; svend.berger@grace.com (S.B.); juergen.nolde@grace.com (J.N.); 2 MolsourceKimya GmbH, Morgenstraße 13, 55257 Budenheim; ty@molsource.de; 3 Institut für Anorganische Chemie und Analytische Chemie, Johannes Gutenberg-Universität, Duesbergweg 10-14, 55128 Mainz, Germany; tremel@uni-mainz.de

**Keywords:** aluminium sulfate, hydrolysis, NMR, REACH

## Abstract

Soluble inorganic aluminium compounds like aluminium sulfate or aluminium chloride have been challenged by the European Chemical Agency to induce germ cell mutagenicity. Before conducting mutagenicity tests, the hydrolysis products in water and in physiological solutions should be determined as a function of the concentration and pH. We used different ^27^Al NMR spectroscopic techniques (heteronuclear Overhauser effect spectroscopy (HOESY), exchange spectroscopy (EXSY), diffusion ordered (DOSY)) in this work to gain the information to study the aluminium species in solutions with Al_2_(SO_4_)_3_ concentrations of 50.0, 5.0, and 0.5 g/L and their pH and time dependent transformation. At low pH, three different species were present in all physiological solutions and water: [Al(OH)_n_(H_2_O)_6 − n_]^(3 − n)+^ (*n* = 0–2), [Al(H_2_O)_5_SO_4_]^+^, and [Al_2_(OH)_2_(H_2_O)_8_]^4+^. Increasing pH reduced the amounts of the two monomer species, with a complete loss at pH 5 for solutions with a concentration of 50.0 g/L and at pH 4 for solutions with a concentration of 5.0 g/L. The dimer species [Al_2_(OH)_2_(H_2_O)_8_]^4+^ is present in a pH range between 3 and 6. Less symmetric oligomeric and probably asymmetric aluminium species are formed at pH of 5 and 6. The pH value is the driving force for the formation of aluminium species in all media, whereas the specific medium had only minor effect. No conclusive information could be obtained at pH 7 due to signal loss related to fast quadrupole relaxation of asymmetric aluminium species. A slight reduction of the content of the symmetric aluminium species due to the formation of oligomeric species was observed over a period of 6 weeks. Reference ^27^Al NMR experiments conducted on saturated water solutions of AlCl_3_ and those with a concentration of 50 g/L show that the type of salt/counter ion at the same concentration and pH influences the hydrolysis products formed.

## 1. Introduction

The REGULATION (EC) No 1907/2006 “REACH” (Registration, Evaluation, and Authorization of Chemicals) [1] dated 18 December 2006 can be considered one of the most complex legislation issued by the European Union [2]. One of the major purposes of the REACH regulation is to improve and secure a high level of protection of human health and the environment, and to enhance the alternative (non-animal based) test methods to determine the hazards of chemical substances. Manufacturers and importers of chemical substances are accountable to gather substance relevant information on physical chemical properties, toxicity, and eco toxicity, which have to be reported through a technical dossier to the European Chemical Agency (ECHA).

Information on the toxicity, especially for humans, shall be generated either with methods other than vertebrate animal tests, through qualitative, quantitative structure-activity relationship models, or based on information from structurally related substances wherever possible [3]. If such investigations are performed on inorganic substances, the behavior in water or water-based solutions has to be determined. Therefore, it is critical to identify the hydrolysis products as a function of the pH and confirm their stability over time in water or water-based solutions.

One of the key concerns of REACH beside data generation and evaluation is the identification of potential CMR effects (carcinogenity, germ cell mutagenicity, and toxicity for reproduction) of substances. This is done by using standardized and internationally accepted test methods, i.e., following the OECD (Organisation for Economic Co-operation and Development) test method guidelines. According to the Guidance on Information Requirements and Chemical Safety Assessment [4], tests to determine mutagenicity are carried out in vitro [5,6,7,8] or in vivo if no non-testing methods such as structure-activity relationship (SAR), quantitative structure-activity relationship (QSAR) [9], or read-across approaches are available.

The Fraunhofer Institute for Toxicology and Experimental Medicine published, in 2017, a final report on a project on behalf of the Federal Institute for Occupational Safety and Health [10], where the solubilities of six different inorganic materials (nano SiO_2_, μ-TiO_2_, nano-TiO_2_, μ-Zr_2_O_3_, Eu_2_O_3_, μ-BaSO_4_) in two different artificial lung fluids (Gamble’s solution [11] and artificial lysosomal fluid [12]) were compared at pH 7.4 and 4.5. The purpose of the study was to define a method to identify potential granular biopersistent particles. One important conclusion was that the solubility varies not only from material to material but may differ even for water-based biological media, for example, when complexation or hydrolysis equilibria are involved. The prediction of the solubility of an inorganic compound in non-innocent solvents is difficult. As solubility with respect to inorganic compounds is associated with a change of structure when a substance comes into contact with water or with biological media, structural investigations are important to derive chemical and structural analogies. These investigations will allow a better prediction of how the structure of substances will change before being tested in in vitro or in vivo.

Soluble inorganic aluminium compounds like aluminium sulfate or aluminium chloride are challenged by ECHA to induce germ cell mutagenicity in 2015 [13]. Read-across approaches taken by industry to demonstrate the similarity of the soluble aluminium compounds towards the less soluble aluminium hydroxide were not accepted because of different solubility and, thus, the biological availability was expected to be different. While it is well known that soluble aluminium compounds like aluminium sulfate form insoluble aluminium hydroxide under neutral conditions, specific analytical methods have to be applied to determine the structure of the aluminium species in biological test media for mutagenicity tests. To conclude on these aluminium species in biological test media used for in vitro mutagenicity tests, commercially produced aluminium sulfate was used for this purpose. A literature review was performed to compare published results with the new data using industrial grade aluminium sulfate dissolved in water to a final concentration typically used in in vivo test methods.

Aluminium sulfate Al_2_(SO_4_)_3_ is used in the form of (Al_2_(SO_4_)_3_·16H_2_O, or more generally alums AM(SO_4_)_2_·12H_2_O, where A is a monovalent cation (e.g., K^+^ or [NH_4_]^+^) and M is a trivalent cation (e.g., as Al^3+^), as a flocculating agent for drinking water purification and waste water treatment. Al^3+^ is added to water to irreversibly destabilize stable suspensions (formed by charged particles) and to induce aggregation with the consecutive precipitation of the aggregates [14]. The mechanism of the flocculation is related with the positive charge of Al^3+^,which reduces the repulsion forces between the electric double layers surrounding the dispersed particles and ions until a critical “zeta-potential” is reached and precipitation occurs [15]. In water, aluminium sulfate hydrolyses to form—depending on the pH—different aquo acid species [16,17]. The process is considered a hydrolysis-polymerization with the formation of distinct complexes at low pH values which develop into polynuclear hydroxo-Al complexes and polymers at neutral pH. There is still no agreement on the mechanism of this process, although a variety of analytical techniques have been used to characterize and quantify polynuclear hydroxyl Al species [18]. The well-known larger aluminum hydroxide molecule is the “cage-like” [Al_13_O_4_(OH)_24_(H_2_O)_12_]^7+^ Keggin-Al_13_ polycation, which was isolated in the form of its sulfate and selenate salts [19] with a Keggin-type structure resembling that of the 12-phosphotungstic acid [20]. The associated model, developed in the 50 s both experimentally [21,22] and theoretically [23], assumes that hydroxylated Al^3+^ undergoes changes from monomeric to polymeric species according to the hexameric ring model. After the formation of the largest gibbsite-fragment of Al_54_(OH)_144_^18+^ in the sol-state, a gel precipitation of Al(OH)_3_ having the sheet structure of gibbsite occurs [24]. On the other hand, the “Cage-like” model assumes that Al^3+^ in solution exists only as (hexaaquo) monomer, dimer, Keggin-Al_13_ structure (AlO_4_Al_12_(OH)_24_(H_2_O)_12_^7+^) and larger polymerized Al species [24,25]. However, both models cannot explain the formation of various (metastable) polymeric aluminium species.

^27^Al NMR spectroscopy is widely applied in analysing the hydrolysis of aluminium salt solutions [24,26,27]. Such studies are mostly related with more concentrated solutions and forced hydrolysis due to the lower sensitivity of the NMR spectroscopy compared to other methods [26]. It was found [24,28,29,30] that in aqueous solutions containing Al^3+^, over a wide range of pH values, only highly symmetric or moderately distorted complexes could be monitored. These species include the hexa-coordinated monomer [Al(OH)_n_(H_2_O)_6 − n_]^(3 − n)+^ (n = 0–2) with ^27^Al signals in the shift range of 0–4 ppm and a number of tetra-coordinated AlO_4_ in the ε- (63 ppm) and γ-isomer (76 ppm) tridecamers [AlO_4_Al_12_(OH)_24_(H_2_O)_12_]^7+^, the central tetra-coordinated AlO_4_ in [Al_2_O_8_Al_28_(OH)_56_(H_2_O)_26_]^18+^ at 70 ppm, and the tetra-coordinated anion Al(OH)_4_^−^ at 80 ppm. A number of other ^27^Al resonances were also observed in the range typical for hexa-coordinated aluminium being, however, significantly broadened and assigned as the dimer [Al_2_(OH)_2_(H_2_O)_8_]^4+^ and the respective trimer with shifts of 2 to 13 ppm [27]. 

Here we report an ^27^Al NMR study on the hydrolysis products of Al_2_(SO_4_)_3_ in four different physiological media as a function of concentration and pH value. At lower pH values it was possible to identify the hydrolysis products using solution NMR. At neutral pH, no signal was detected due to the very fast quadrupole relaxation of Al in a non-symmetric environment. A broad band solid state NMR spectrometer at very low magic angle spinning (MAS) and an excitation range of ca. 3.2 MHz was used to check for the existence of very broad ^27^Al signals, however, with no success.

The distribution of the resulting hydrolysis products at different pH values in water were compared for three different concentrations. Based on the conducted ^27^Al NMR experiments as well as earlier studies we identified most of the hydrolysis products. We observed the presence of three different aluminum species at pH 3 in all physiological media and water. The dominant species is the highly symmetric [Al(OH)_n_(H_2_O)_6 − n_]^(3 − n)+^ (n = 0–2) with only small amounts of [Al(OH)_n_(H_2_O)_5 − n_SO_4_]^+^ (n = 0–2) and the dimer [Al_2_(OH)_2_(H_2_O)_8_]^4+^ being present besides the major component. At pH 4, the situation changes significantly. The main hydrolysis product is still the symmetric monomer, but the content of the less symmetric dimer characterized by a ^27^Al NMR shift of ca. 5 ppm [16] increased compared to that at a pH value of 3. Changing the pH to 5 and 6 results in the appearance of new significantly broadened and overlapping ^27^Al NMR resonances detected in lower field in the range of ca. 5–12 ppm. The signals can be identified as, most likely, octahedral complexes with lower symmetry including dimer, trimer, and higher oligomers. At pH 7, no aluminium signal was detected. The formation of a residue indicates the formation of colloidal hydrated alumina. The solution samples remained stable for 6 weeks at ambient conditions with only minor changes in the ratio of the hydrolysis products that slightly shift towards the formation of oligomeric species.

## 2. Results and Discussion

Aqueous solutions of Al_2_(SO_4_)_3_ in the pH range from 3 to 7 with three different concentrations (0.5, 5.0, and 50.0 mg/L) were prepared in different physiological media (Table 1) and studied by ^27^Al NMR spectroscopy.The pH was adjusted with the buffers as compiled in Table 2 (Materials and Methods). Roswell Park Memorial Institute Medium commonly referred to as RPMI 1640 (L1) and Dulbecco’s Modified Eagle Medium (DMEM; L2) are two of the commonly used cell culture media for growing mammalian cells. To allow a precise comparison of potential side reactions, both media are used with and without additives. RPMI 1640 was selected in pure form and added with Penicillin, Streptomycin, and Phytohemagglutinine. The enriched medium is referred in Table 1 as L3. Penicillin, Streptomycin, and Phytohemagglutinine are used as inhibition agents to minimize growth of microorganisms. The added Horse serum in DMEM is a typical promoter of proliferation of the cells. The enriched DMEM medium is referred as L4. L4 is the typical medium for the in vitro gene mutation test using the hypoxanthine-guanine phosphoribosyltransferase (HPRT) genes [6].

For all media, the solutions with concentrations 0.5, 5.0, and 50.0 g/L at pH 3 and 4 were clear, whereas those at pH 5 and 6 were turbid with an incipient formation of a precipitate with time. In all samples with pH 7, a gel-like precipitate was observed after the initial dissolution. Before each NMR experiment, the solutions were homogenized by shaking the NMR tube.

### 2.1. Identification of the Hydrolysis Products of Al_2_(SO_4_)_3_ at Different pH Values

^27^Al NMR spectroscopy was used to identify and characterize the hydrolysis products of aluminium sulphate. The ^27^Al nucleus is magnetically active (I = 5/2) with good receptivity relative to ^13^C (D = 1170) and 100% natural abundance. Being a quadrupole nucleus, it interacts with the external magnetic field and the electric field gradient generated by the surrounding environment [31]. The ^27^Al NMR spectra are broadened due to the quadrupole interaction. They remain, however, very informative as the shift range—from ca. −100 to 300 ppm—is large. Furthermore, there is a direct correlation of the chemical shift and the coordination number of the aluminium cation [26,27]. The hexa-coordinated aluminium cations are mostly shielded and resonate in higher fields (shift range −30 to 30 ppm relative to the reference Al(H_2_O)_6_^3+^ at 0 ppm), whereas a low field shift was observed for the penta-coordinated aluminium species. The tetra-coordinated aluminium species is characterized by the lowest field shifts compared to the former two classes. The drawback of ^27^Al NMR spectroscopy, although it is widely applied, is that it can detect only aluminium in a symmetric environment. The loss of symmetry, as in the case of large complexes and polymeric species, leads to a fast quadrupole relaxation. Therefore, Al spectra of such species are broadened beyond detection in NMR spectroscopy.

**Figure 1:**
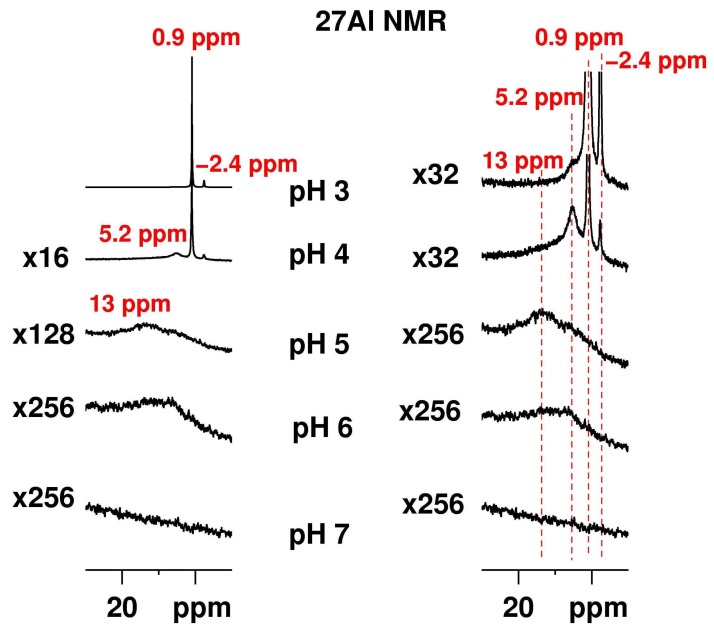
^27^Al NMR solution spectra of Al_2_(SO_4_)_3_ in L1 medium recorded at pH 3 to 7 and scaled to the signal with the highest intensity at 0.9 ppm (**left**) with the spectral region characteristic for the hexa-coordinated Al magnified (**right**). All spectra were recorded without ^2^H spin lock as no deuterated solvent was used. For all spectra, the background ^27^Al signal due to the glass of the NMR tube was recorded in a separate experiment with the same number of scans and receiver gain and subtracted from the spectrum of the respective sample.

Al_2_(SO_4_)_3_ was dissolved in concentrations of 0.5 g/L, 5.0 g/L, and 50 g/L in four different physiological media, L1–4, and water for comparison at pH values ranging from 3 to 7. This concentration range is typical for in vitro testing. All recorded ^27^Al NMR spectra for the different physiological media were similar for the same pH. Therefore, only representative spectra of Al_2_(SO_4_)_3_ dissolved in the L1 medium in a concentration of 50 g/L (Figure 1) are discussed. 

In the ^27^Al NMR spectrum of Al_2_(SO_4_)_3_ in L1 medium at pH 3, two resonance signals at ca. 0.9 ppm and at ca. −2.4 ppm with an integral ratio of 9:1 are clearly detected. It should be noted that at these conditions, no peak at ca. −6.7 ppm related with the formation of a bi-sulfato complex [26] was detected. Such a resonance signal has been observed earlier, also in the case of a forced hydrolysis of aluminium through the thermal decomposition of urea for the [Al(H_2_O)_4_(urea)_2_]^3+^ at −4.47 ppm in addition to the signal of the monosubstituted urea complex [Al(H_2_O)_5_(urea)]^3+^ at −2.67 ppm [32].

In the current study, a shoulder at ca. 5 ppm at the base of the peak related with the hexa-aquo complex at 0.9 ppm being observed. The shifts of three detected ^27^Al resonances are within the shift range of hexa-coordinated Al^3+^ [26,27]. We attribute the signal at 0.9 ppm to the presence of [Al(H_2_O)_6_]^3+^ in solution [16]. It is worth mentioning that the measurements were conducted without ^2^H spin lock. Therefore, a slight deviation from the 0 ppm value is not unexpected. Hexa-coordinated aluminium nuclei, which possess one [SO_4_]^2−^ anion according to some sources in the first [16,33] or the second [34] coordination shell, resonate slightly up-field.

To check the presence of a coordination shell of water molecules around the Al^3+^ cations, 2D ^27^Al heteronuclear Overhauser effect spectroscopy (HOESY) experiments with mixing times of 10, 30, 50, and 100 ms and 1 s were carried out (Figure S1, Supplementary Information).The HOESY experiment brings information about the spatial connectivity between heteronuclear spins, i.e., the signals in the spectrum corresponding to the through space correlation between dipolar coupled spins situated within a distance smaller than 5 Å. 

**Figure 2:**
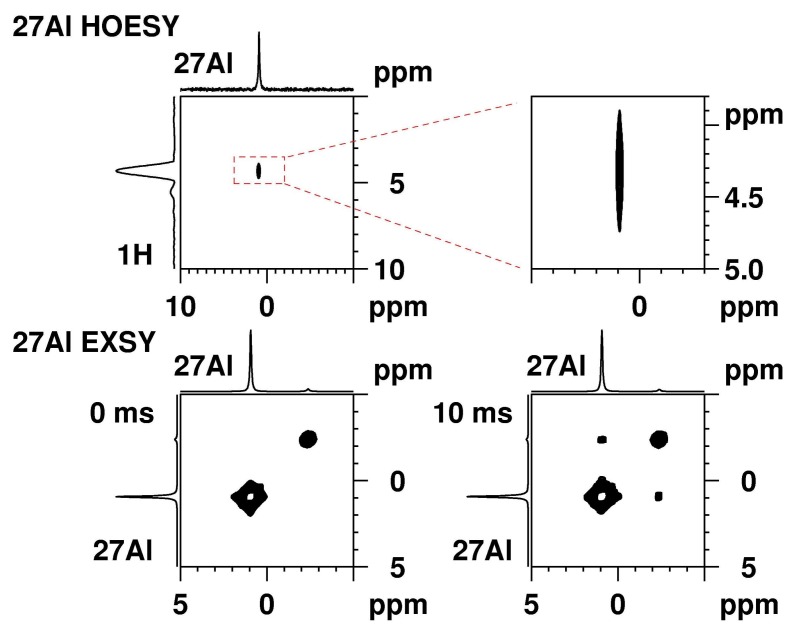
(**top**) ^27^Al heteronuclear Overhauser effect spectroscopy (HOESY) spectrum of Al_2_(SO_4_)_3_ recorded in L1 solution at pH 3 with 30 ms mixing time (**left**) with the region characteristic for hexa-coordinated aluminium (magnified, **right**); (**bottom**) ^27^Al exchange spectroscopy (EXSY) spectra recorded with 0 ms mixing time (**left**) as a reference spectrum and 10 ms mixing time (**right**).

Figure 2 (top) presents the ^27^Al HOESY spectrum of Al_2_(SO_4_)_3_ in solution L1 at pH 3 recorded with a mixing time of 30 ms. The intense correlation signal at the Al shift is characteristic for octahedral aluminium in [Al(H_2_O)_6_]^3*^ and water molecules at 4.3 ppm. This intensive signal was detected also in all ^27^Al HOESY spectra recorded with 10 ms, 30 ms, 100 ms, and 1 s mixing times. Water molecules are present in close vicinity, i.e., in the coordination shell of the aluminium species. It should be noted that at longer mixing times, there should be a contribution coming also from the water molecules of the second coordination sphere and from the solution. A correlation signal for the low intensity resonance at −2.4 ppm was detected in the spectrum recorded with 1 s mixing time. Also, a through space connectivity with water molecules was confirmed. However, a water coordination of this species could not be confirmed unequivocally. There are indications that the species responsible for the signal at 5.2 ppm (Figure S1), and which appears as a shoulder in the ^27^Al spectrum, has water molecules in close proximity as well. It is, however, difficult to confirm this beyond doubt, as the signal has only low intensity and overlaps with the base of the resonance of [Al(H_2_O)_6_]^3+^.

^27^Al T_1_ relaxation measurements conducted by applying the inversion recovery method brought further information about the observed Al species. The spin-lattice relaxation times determined for the signals at 0.9 and −2.4 ppm were 52 ms and 48 ms, respectively. A comparable T_1_ relaxation time was observed earlier for a 1.5 M water solution of AlCl_3_ [35]. Such relatively long T_1_ relaxation times for the quadrupole aluminium nucleus imply a symmetric environment. This is consistent with the suggested octahedral coordination for both complexes. Additionally, these comparable T_1_ times suggest a chemical exchange between these two Al species. The Al^3+^ ions typically carry ligands; therefore, a chemical exchange of aluminium ions is not possible. However, the surrounding ligands may exchange, which may lead to different chemical environments, i.e., different chemical shifts for the ^27^Al nuclei. ^27^Al exchange spectroscopy (EXSY) NMR could be used as evidence for an equilibrium ligand exchange between Al^3+^ species according to

(1) $$\begin{array}{ccc} {\left[\mathrm{Al}{\left(H_{2}O\right)}_{6}\right]}^{3+} & \overset{K_{\mathrm{AB}}}{\underset{K_{\mathrm{BA}}}{⇌}} & {\left[\mathrm{Al}{\left(H_{2}O\right)}_{5}{\mathrm{SO}}_{4}\right]}^{+}\\ A & & B \end{array}$$

In addition, the exchange rate constants for both reactions could be estimated [16,26]. They were determined at the coalescence point of both signals by measuring variable temperature (VT) 1D NMR spectra [16]. A drawback of this method is that the viscosity is reduced at higher temperatures, which affects the rotational correlation time of the respective complexes. It has an immediate effect on the line width of the aluminium signal as well. In addition, high temperatures may affect the lifetime of the complexes. 

We conducted ^27^Al exchange spectroscopy (EXSY) on the sample in L1 medium at pH 3 at ambient conditions with mixing times ranging from 1.5 ms to 50 ms (Figure S2). Figure 2 (bottom) shows representative ^27^Al EXSY spectra measured with 0 ms (reference experiment) and 10 ms mixing times. The spectrum recorded with 10 ms mixing time reveals cross-correlation signals between the 0.9 and the −2.4 ppm ^27^Al resonances. These cross-peaks have the same phase as the diagonal signals, which confirms a chemical exchange between both aluminium species. Such correlation signals are detected in all spectra with mixing times below 50 ms. Longer mixing times up to 1 s (spectra not presented) lead to signal decay due to relaxation.

The exchange rate constants k_AB_ and k_BA_ for the reversible reaction in Equation (1) were derived from the intensities in two consecutive EXSY experiments recorded at the same conditions—the reference experiment with 0 s mixing time and the EXSY experiment with 30 ms mixing time (using the EXSYCALC program from Mestrelab) [36,37]. At 292 K, the rate constant for the formation of the sulfo-complex B was determined as k_AB_ = 0.217 s^−1^, whereas that for the formation of the hexaaquo-Al-complex A was k_BA_ = 81.669 s^−1^.

In order to characterize the motional behavior of both Al species, we applied diffusion ordered spectroscopy (DOSY). In this experiment, the diffusion coefficient is correlated to the chemical shift by applying a pulsed gradient field of varying strength to obtain an exponential decay of the ^27^Al-resonances as a result of the molecules diffusing out of the excitation range. The ^27^Al signal intensity is attenuated depending on the diffusion time. It is worth mentioning that the signal decay and increasing gradient strength do not follow the “standard” exponential relation. Even a strong gradient field did not attenuate the Al-signal significantly. The obtained diffusion coefficients from the diffusion analysis, 2.4 × 10^−11^ m^2^/s for the [Al(H_2_O)_6_]^3+^ complex at 0.9 ppm and 3.4 × 10^−11^ m^2^/s for [Al(H_2_O)_5_SO_4_]^+^ at −2.4 ppm, are too high for small complexes. Such values might describe complexes with a large coordination shell (affected by chemical exchange) or clusters.

Significant changes were observed in the ^27^Al NMR spectrum of Al_2_(SO_4_)_3_ dissolved in L1 when the pH was increased to 4 (Figure 1). The intensity of the signals at 0.9 ppm related to [Al(H_2_O)_6_]^3+^ and the one at −2.4 ppm related to [Al(H_2_O)_5_SO_4_]^+^ are dramatically reduced to less than 5% of the intensity in the spectrum at pH 3. The resonance at 5.2 ppm assigned to the [Al_2_(OH)_2_(H_2_O)_8_]^4+^ dimer species gains intensity to about 50% compared to that of the resonance at pH 3. A broad shoulder appears at lower fields. The ^27^Al intensity corresponding to this shoulder is at least partially associated with the trimer [Al_3_(OH)_4_(H_2_O)_10_]^5+^ [24]. Considering the reported shifts of the ^27^Al resonance to a lower field with increasing nuclearity of the species in solution, higher oligomers may contribute to this intensity as well. We observe an overall “loss” in Al signal intensity. This phenomenon has been noted for ssNMR [24] and is related with NMR resonances from Al sites having low symmetry. The result is a large electric field gradient which can broaden the NMR signals beyond detection. Several methods have been proposed to quantify this “invisible aluminium” contribution. Some of them include the shift of the measurements to higher magnetic fields using fast and ultra-fast magic angle spinning (MAS) [38], while others rely on short pulse angles (of about 15 degree) on the resonance to excite quantitatively the principle (−1/2 + 1/2) transition [39]. Methodological developments like different echo techniques [40], Multiple-Quantum Magic-Angle Spinning (MQMAS) [40,41], or TRAnsfer of Populations in DOuble Resonance (TRAPDOR) [42] were proposed as well. In solution, in addition to the large electric field gradients, an important role may also be played by the slow tumbling times for large complexes in viscous media [24]. An elegant way to solve the problem of quantifying the “invisible aluminium” has been proposed [43]. So far, the NMR “invisible” Al species have been identified with the help of electrospray ionization mass spectroscopy (ESI-MS) from the hydrolysis of AlCl_3_ [44,45] at pH 4 to 6.4.

The 2D experiments conducted at pH 4 were partially successful due to the fast quadrupole relaxation of the asymmetric Al sites. It was possible to determine the T_1_ relaxation times for the main resonances at −2.4 ppm (31 ms), 0.9 ppm (18 ms), and at ca. 5.6 ppm (1.6 ms). The reduction of the T_1_ relaxation time of the signal at 0.9 ppm indicates that [Al(H_2_O)_6_]^3+^ is transformed into higher oligomers and polymers. At the same time, the intensity loss for [Al(H_2_O)_5_SO_4_]^+^ appeared related to the transformation to the hexa-aquo complex, which shifts the equilibrium to the polynuclear species. 

At pH 5 and 6, the solution is no longer clear. Incipient precipitation was observed, which is related to a change in the degree of hydrolysis (i.e., the [OH^−^]/[Al^3+^] ratio), which describes the extent to which the acidic buffer capacity of pure salt solutions is consumed [46]. For [OH^−^]/[Al^3+^] ≈ 2.5, the solution is fully hydrolyzed and precipitation occurs [17,46]. 

At pH 5, the critical [OH^−^]/[Al^3+^] ratio has been exceeded, and the aluminium content is distributed between the precipitate, the “invisible aluminium”, and the dissolved species. Information about the latter was obtained from the ^27^Al NMR spectrum. The ^27^Al resonances for the [Al(H_2_O)_6_]^3+^ monomer (0.9 ppm), the [Al_2_(OH)_2_(H_2_O)_8_]^4+^ dimer (5.2 ppm), and the sulfo complex [Al(H_2_O)_5_SO_4_]^+^ (−2.4 ppm) were no longer detected, and a broad signal covering a shift range of ≈30 ppm was observed. This shift range is still related to hexa-coordinated aluminium, the broadening being due to species with some asymmetry in their environment.

At pH 7, a significant amount of Al(OH)_3_ sol precipitated [27]. No ^27^Al NMR resonances could be detected due to extreme signal broadening. ^27^Al NMR experiments were conducted on samples dissolved in L1 on a broad band solid state NMR spectrometer, exciting a bandwidth of ca. 3.2 MHz. The recorded spectra (not shown) did not reveal additional signals. 

### 2.2. Comparison of the ^27^Al Signals in the Spectra Recorded for Different Media

**Figure 3:**
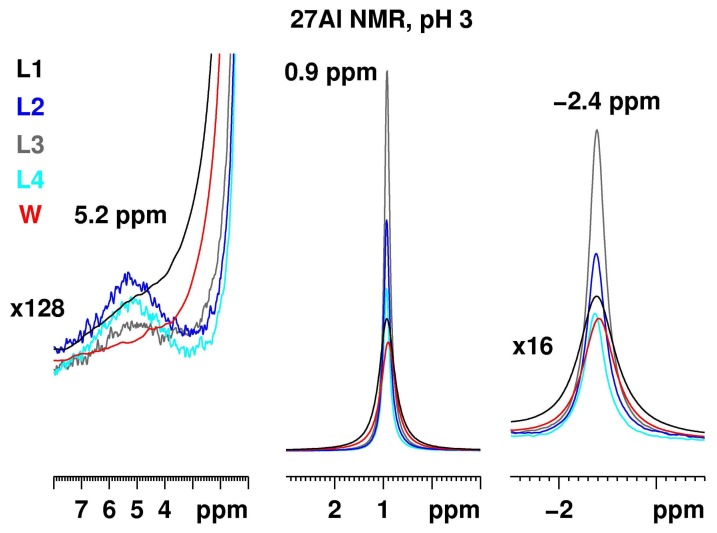
^27^Al NMR of Al_2_(SO_4_)_3_ with a concentration of 50.0 g/L at pH 3 recorded at identical conditions for media L1–4 and water. The three signals at 5.2 ppm for the dimer, at 0.9 ppm for the aluminium hexahydrate and at −2.4 ppm for the [Al(H_2_O)_5_SO_4_]^+^, are scaled relative to the most intense resonance at 0.9 ppm.

Figure 3 presents the ^27^Al NMR spectra of Al_2_(SO_4_)_3_ solutions with a concentration of 50.0 g/L at pH 3 recorded at the same conditions in the media L1–4 and water. Three signals at 5.2 ppm for the dimer, at 0.9 ppm for [Al(H_2_O)_6_]^3+^ and at −2.4 ppm for [Al(H_2_O)_5_SO_4_]^+^, are detected in all spectra. The signals are scaled relative to the one at 0.9 ppm. No significant intensity differences are observed. Thus, the main effect on the spectra is related to the pH of the solutions.

The ^27^Al spectra of Al_2_(SO_4_)_3_ dissolved in RPMI 1640 medium (L1) and water are very similar in terms of signal intensities and ratios. The resonances at 5.2 ppm are detected as shoulders of the main peak at 0.9 ppm. The addition of antibiotics and the lectin phytohemagglutinin (PHA consisting of two closely related proteins PHA-L and PHA-E, which agglutinate leucocytes and erythrocytes, respectively) in the RPMI 1640 medium slightly enhances the formation of more symmetric species at pH 3.

In the ^27^Al NMR spectra of Al_2_(SO_4_)_3_ solutions in DMEM medium, the three Al resonances (mentioned above) were observed. The signal intensities and the respective signal ratio of the signals at 0.9 and −2.4 ppm are comparable to the RPMI 1640 medium. Clearly, the DMEM medium promotes dimer formation at the expense of the [Al(H_2_O)_6_]^3+^ hexaaquo and the [Al(H_2_O)_5_SO_4_]^+^ complex. This may be related with a slight increase of the pH value. Additionally, the intensities in the spectrum in the L4 medium are lower than those in the DMEM medium. The addition of the antibiotics has only a minimal influence on the ^27^Al NMR spectrum. The addition of horse serum to the DMEM medium (L4) is responsible for a decrease of the ^27^Al intensity compared to the pure DMEM medium. It should be noted that serum albumin (including horse serum), the most abundant plasma protein in mammals, is characterized by an extraordinary ligand [47] and metal [48] binding capacity. Therefore, it is not surprising that the presence in the solution of another binding functionality could promote formation of non-symmetric (including hexa-coordinated) aluminium complexes. Moreover, it has been confirmed by a number of techniques such as spectrophotometric titration, ultrafiltration, dialysis and gel chromatography [49] that A1^3+^ in serum is highly protein bound to both human serum albumin (HAS) and human serum transferrin (HSTF), at the protein concentrations found in blood plasma. Furthermore, pH dependence of the Al^3+^ binding has been observed with maximal binding at neutral pH [49]. Based on those results, it was suggested that HSA and HSTF may serve as carriers in the biological transport of A1^3+^ in blood plasma [49 and references cited therein].

Considering the relatively small amount of horse serum at this aluminium sulfate concentration and the weaker binding of Al^3+^at pH 3, it is not surprising that there is no striking difference between the RPMI and water solutions. A more pronounced influence on the NMR spectra is expected at lower Al_2_(SO_4_)_3_ concentrations and higher pH values. No precipitation was observed in all five samples.

**Figure 4:**
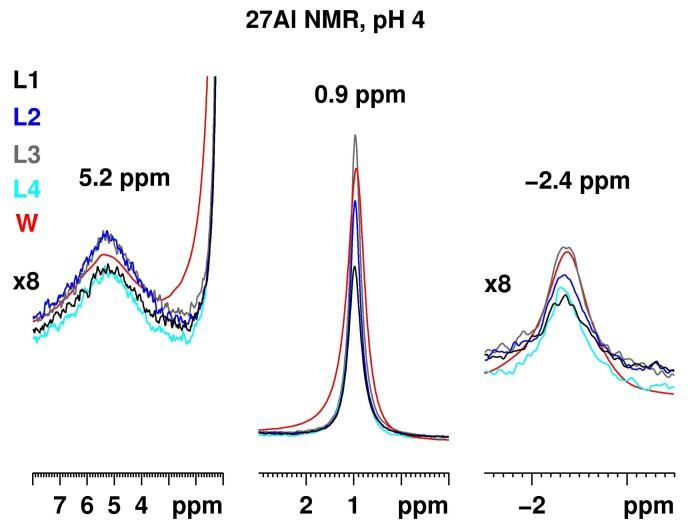
^27^Al NMR of Al_2_(SO_4_)_3_ with a concentration of 50.0 g/L at pH 4 recorded at the same conditions for the different media L1–4 and water. The three signals at 5.2 ppm for the dimer, at 0.9 ppm for the aluminium hexahydrate cation and at −2.4 ppm for the [Al(H_2_O)_5_SO_4_]^+^, are scaled relative to the most intense resonance at 0.9 ppm.

Figure 4 presents the ^27^Al NMR spectra of Al_2_(SO_4_)_3_ with a concentration of 50.0 g/L at pH 4 recorded at the same conditions in the media L1–4. The three aluminium resonances at 5.2 ppm for the dimer, at 0.9 ppm for the aluminium hexaaquo-complex and at −2.4 ppm for the [Al(H_2_O)_5_SO_4_]^+^, are present in all media. The signal intensities have, however, changed significantly, confirming the strong pH dependence of the Al spectra. There are some slight differences related with the constituents and their behavior at pH 4. The most intense resonances were observed in the spectrum of aluminium sulfate dissolved in the L3 medium.

**Figure 5:**
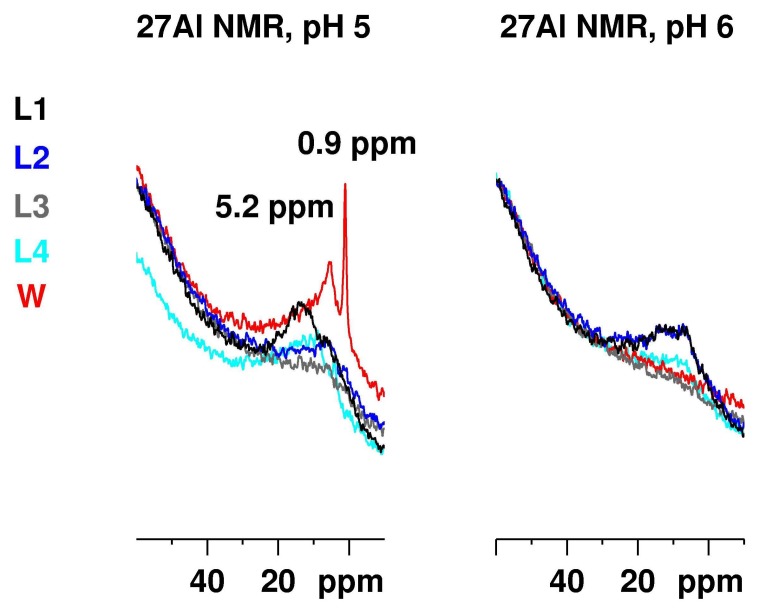
^27^Al NMR of Al_2_(SO_4_)_3_ with a concentration of 50.0 g/L at pH 5 (**left**) and 6 (**right**) recorded at the same conditions for the different media L1–4 and water. The signals at −2.4 ppm for the [Al(H_2_O)_5_SO_4_]^+^ are no longer visible for all media. The signal at 0.9 ppm for the aluminium hexahydrate is detected only in the case of the water solution.The resonance at 5.2 ppm for the dimer is observed clearly in the water solution and significantly reduced in L1 medium. A new broadened ^27^Al resonance at ca. 12 ppm appears instead. At pH 6, this broad signal remains only in the RPMI and DMEM media.

Figure 5 presents the ^27^Al NMR spectrum of Al_2_(SO_4_)_3_ with a concentration of 50.0 g/L at pH 5 (left) and 6 (right) recorded at the same conditions in the media L1–4 and water. In all spectra the intensity related with [Al(H_2_O)_5_SO_4_]^+^ vanished and that associated with the monomer [Al(H_2_O)_6_]^3+^ was visible only for the water solution at pH 5. Some intensity, associated with the dimer, remained for the water solution and—to a smaller extent—in the L1 media at pH 5. In addition, a new significantly broadened signal appeared with a maximum at ca. 12 ppm. Surprisingly, the Al signals in the L3 medium that were most intense at pH 3 and 4, decayed at pH 5. A precipitate was formed in all solutions (except for the aqueous solution).

At pH 6, precipitation occurred in all five solutions. No intensity was detected for the L3 and the water solutions. Only broad Al resonances in the media L1 and L2 were detected, where no additives were used. The same signal, but with reduced intensity, was observed in the L4 media as well.

At pH 7, no signal was detected in the Al NMR spectra as the Al^3+^ cations are in an asymmetric environment due to the formation of the Al(OH)_3_ sol.

Figure S3 presents the ^27^Al NMR spectra of Al_2_(SO_4_)_3_ with a concentration of 5.0 g/L in the pH range 3–7 recorded at the same conditions for media L1–4 and water. At pH 3, the spectra in all media were comparable as detected resonances with the spectra at higher concentrations. The three signals at 5.2 ppm for the dimer [Al_2_(OH)_2_(H_2_O)_8_]^4+^, at 0.9 ppm for [Al(H_2_O)_6_]^3+^ and at −2.4 ppm for the [Al(H_2_O)_5_SO_4_]^+^, were present. At pH 4, the resonance at −2.4 was no longer visible for all media. The signal for [Al(H_2_O)_6_]^3+^ was still observed in the water solution with dramatically reduced intensity. Instead, a new broadened peak appeared at ≈12 ppm. Such a resonance is related with hexa-coordinated aluminium in an asymmetric environment, most probably due to the formation of oligomeric species. At pH 4 to 6, the dominating signal is that associated with oligomeric species. No signal was detected for all samples at neutral pH. Thus, we conclude that at lower concentrations, the formation of oligomeric species occurs at pH 4–6, i.e., the condensation equilibrium [Al(H_2_O)_6_]^3+^ = Al(OH)_3_(H_2_O)_3_ + 3H^+^ is shifted to the right. 

The spectra of the samples at the lowest concentration of 0.5 g/L were recorded with 1024 scans to enhance the aluminium signal intensity (Figure S4). Even at these conditions, meaningful spectra could be measured only for pH 3 in water as all three characteristic signals for this pH observed at the higher concentrations were detected. For the L1 medium, the signal at 0.9 ppm was still visible. It is expected that the main hydrolysis product/s in the pH range 4–6 remain as hydrated oligomeric aluminium species.

To evaluate the influence of the type of aluminium salt in relation to the type of counter ion on the ^27^Al NMR spectra in aquous solutions as a function of pH, we recorded representative spectra of AlCl_3_ and compared it to the spectra of Al_2_(SO_4_)_3_. Figure S5 presents the spectra of the 50 g/L water solutions of AlCl_3_ and Al_2_(SO_4_)_3_ as well as Al_2_(SO_4_)_3_ water solution enriched with 1% penicillin. The signals of the hexaaquo-complex [Al(H_2_O)_6_]^3+^ and the dimer [Al_2_(OH)_2_(H_2_O)_8_]^4+^ were detected in all spectra except the AlCl_3_ solution at pH 5. The resonances for the [Al(H_2_O)_5_SO_4_]^+^ were visible in the spectra of aluminium sulfate at pH 3 and 4. At the same, conditions chlor-containing-aquo-complexes were not observed in the aluminium chloride solutions independent of the concentration (Figure S6). Clearly, the stability of the [Al(H_2_O)_5_SO_4_]^+^ complex is higher compared to the respective chlor-containing analog. Moreover, it is known that N- and O-containing hard Lewis bases (as (SO_4_)^2−^) form stable complexes with light s- and p-metal cations (such as Al^3+^). The type of salt/counter ion at the same concentration and pH has an effect on the hydrolysis products formed. The presence of penicillin resulted in a slight reduction of the intensity of the monomer signal at pH 5.

It should be noted that in all Al_2_(SO_4_)_3_ solutions, independent of the concentration, no signal for the A13 Keggin polycomplex was observed. However, the ^27^Al NMR spectrum recorded for a saturated solution ofAlCl_3_ in water for comparison (Figure S6) contained the resonance for the tetra-coordinated central AlO_4_ in the ε-isomer (64 ppm) and in the polycation [AlO_4_Al_12_(OH)_24_(H_2_O)_12_]^7+^ at pH 4 and 5. This fact confirmed that the type of salt/counter ion at the same concentration and pH influences the hydrolysis products formed.

### 2.3. Stability of the Al_2_(SO_4_)_3_ Solutions

To check the stability of the Al_2_(SO_4_)_3_ solutions we have chosen representative samples with a concentration of 50.0 g/L in the pH range from 3 to 6 in L1 media. The samples were kept in NMR tubes at ambient conditions for 6 weeks. Subsequently, ^27^Al NMR spectra were measured under identical experimental conditions using identical datasets. Before each measurement, the samples were homogenized by shaking the NMR tube several times. The recorded NMR spectra shown in Figure S7 were compared with those obtained 6 weeks earlier.

Slight changes in the spectra are due to aging. At pH 3, the intensity of all signals decreased by ca. 8%. At pH 4, a comparable decrease was detected for the signal of the [Al_2_(OH)_2_(H_2_O)_8_]^4+^ dimer, while those associated with [Al(H_2_O)_6_]^3+^ and [Al(H_2_O)_5_SO_4_]^+^ remained unchanged. The most pronounced differences occured at pH 5 and 6, where the broad signal at 12–13 ppm sharpened. We attribute the broadening to the formation of a better defined (but still asymmetric) hexa-coordinated Al species. At pH 7, no signal was detected.

Based on the results for these representative samples, slow changes occur in the solutions of Al_2_(SO_4_)_3_ over a prolonged period of time. These changes are related to the decreasing Al signal intensity of [Al(H_2_O)_6_]^3+^, [Al(H_2_O)_5_SO_4_]^+^, and [Al_2_(OH)_2_(H_2_O)_8_]^4+^ dimers at lower pH and formation of an oligomeric Al species at pH 5 and 6. 

## 3. Materials and Methods

Al_2_(SO_4_)_3_ was obtained from Grace Silica GmbH (Dueren, Germany) and used without further treatment. The medium L1 (Roswell Park Memorial Institute Medium (RPMI 1640)) with stable l-glutamine containing 25 mM 4-(2-hydroxyethyl)-1-piperazineethanesulphonic acid (HEPES), 5.5 g/L NaCl, 5 g/L phenol red, and 2.0 g/L NaHCO_3_ was purchased from Biochrom GmbH and used without further treatment. Medium L2 (Dulbecco’s Modified Eagle Medium (DMEM)) without l-glutamine containing 3.7 g/L NaHCO_3_ and 4.5 g/L d-glucose was purchased from Biochrom and used without further treatment. The medium L3 was prepared from L1 with the addition of 2% phytohemagglutinin, 1% penicillin (10,000 U/mL), and 1% streptomycin (10,000 U/mL). Medium L4 was prepared from L2 with the addition of 5% horse serum, 1% penicillin (10,000 U/mL) and 1% streptomycin (10,000 U/mL). The pH was adjusted with the following buffers:

All ^27^Al NMR experiments were conducted on a Bruker Avance DRX 400 (Bruker Biospin GmbH, Rheinstetten, Germany) NMR spectrometer operating at a ^1^H frequency of 400.31 MHz and ^27^Al frequency of 104.31 MHz, equipped with a 5 mm inverse two channel probe head with z-gradients. All experiments were recorded without ^2^H lock as no deuterated solvent was used. The single pulse excitation experiments were measured with a recycle delay of 1 s, averaging 32 scans. For the ^27^Al NMR experiments with background suppression, 128 scans for the 5.0 g/L and 50.0 g/L concentrations and 1024 scans for the 0.5 g/L concentration were averaged with a recycle delay of 2 s. The inversion-recovery NMR method was used to determine the ^27^Al spin-lattice relaxation times. For the ^27^Al exchange spectroscopy (EXSY) experiments, 2048 data points were recorded in the direct dimension with 1024 increments in the indirect dimension and 16 scans per increment in a phase sensitive mode. The mixing times were set to 0 s (reference experiment) and 1.5, 5, 10, 30, and 50 ms and a recycle delay of 0.5 s was used. The exchange rate constants were determined from the reference experiment and with the 10 ms mixing time using the EXSYCALC software package from Mestrelab. The ^27^Al heteronuclear Overhauser effect spectroscopy (HOESY) experiments were conducted with aluminium detection, recording 4096 data points in the direct dimension with 64 increments in the indirect dimension and 256 scans per increment. The recycle delay was set to 1 s. Stimulated echo sequence with bipolar gradient pulses and a longitudinal eddy current delay was used for the ^27^Al diffusion ordered spectroscopy (DOSY) experiments. The gradient strength was incremented in 16 steps from 2% to 95% of the maximum gradient strength. The diffusion time and the gradient pulse length for the Al_2_(SO_4_)_3_ sample at pH 3 dissolved in L1 medium were 120 ms and 2.8 ms with 2 s recycle delay, respectively. After Fourier transformation and baseline correction, the diffusion dimension of the 2D DOSY spectra was processed using the Bruker Topspin 1.3 software package (2005, patchlevel 8, Bruker Biospin GmbH, Rheinstetten, Germany). The diffusion analysis was performed using the Topspin T_1_/T_2_ relaxation package.

^27^Al NMR reference spectra were recorded for the samples dissolved in L1 medium for all pH values using a broad band solid state Bruker Avance DSX 400NMR spectrometer operating at a frequency of 104.2 MHz. A commercial three channel probe head from Bruker at very low magic angle spinning (MAS) of ca. 2 kHz with no spinning frequency control was used for the experiments. The ^27^Al NMR experiment for the sample with neutral pH was recorded, averaging 30 k scans with 2 s repetition time and 3.2 MHz excitation bandwidth to check for signal contributions from polynuclear hydroxo-Al complexes and polymers.

## 4. Conclusions

In this study, we identified the hydrolysis products of Al_2_(SO_4_)_3_ at three different concentrations of 50.0, 5.0, and 0.5 g/L in four physiological media and water, which are routinely used to conduct mutagenicity tests. Measurements were made at pH values of 3, 4, 5, 6, and 7 to cover the relevant range where Aluminium sulfate hydrolyses. The pH value is the driving force for the formation of the observed aluminium species in all media. The kind of medium used compared to water has only minor influence. At a concentration of 50 g/L and pH values of 3 and 4, three different species were observed in all media: the highly symmetric monomeric [Al(OH)_n_(H_2_O)_6 − n_]^(3 − n)+^ (n = 0–2) as the main product, the dimer [Al_2_(OH)_2_(H_2_O)_8_]^4+^, and the sulfo-containing complex [Al(H_2_O)_5_SO_4_]^+^. Increasing the pH lead to a reduced content of the monomeric species and sulfo-containing complex in favor of an increased dimer content. At pH of 5 and 6, the former two complexes degraded while the latter one transformed into asymmetric hexa-coordinated oligomeric Al species and formed sol-like Al(OH)_3_. At pH 7, no signal was detected due to the formation of sol-like Al(OH)_3_. Lower concentrations promote the formation of oligomeric species in a broader pH range (4 to 6). Al_2_(SO_4_)_3_ concentrations in the range of 0.5 g/L do not provide conclusive information based on NMR spectroscopy due to low signal intensity. The initial conversion of Aluminium sulfate by coming into contact with medium or water at adjusted pH value is formed very fast (within a couple of seconds), later on, the samples remained relatively stable over a period of 6 weeks with slight changes related to a decrease in the content of the monomer, dimer, and sulfate-complex, and oligomeric and asymmetric (most probably polymeric) species were formed instead. Reference ^27^Al NMR experiments recorded for aqueous solutions of AlCl_3_ demonstrate that the type of aluminium salt/counter ion influences the hydrolysis products formed.

## Figures and Tables

**Table 1:** Physiological media used to dissolve the Al_2_(SO_4_)_3._

Medium	Medium
L1	Roswell Park Memorial Institute Medium (RPMI 1640)
L2	Dulbecco’s Modified Eagle Medium (DMEM)
L3	RPMI 1640 with Penicilin, Streptomycin, and Phytohemagglutinine
L4	DMEM with Penicilin, Streptomycin, and Horse Serum for HPRT
W	Distilled Water

**Table 2:** Composition of the buffer solutions used to adjust the pH of the media L1–4.

pH	Composition
3	7.74 g citric acid (waterfree) + 3.49 g NaCl + 206 mL NaOH (c = 0.1 mol/L)
4	10.75 g citric acid (waterfree) + 2.57 g NaCl + 68 mL NaOH (c = 1 mol/L)
5	18.52 g citric acid (waterfree) + 196.4 mL NaOH (c = 1 mol/L)
6	11.46 g citric acid (waterfree) + 159.6 mL NaOH (c = 1 mol/L)
7	3.52 g KH_2_PO_4_ + 7.26 g Na_2_HPO_4_ · 2H_2_O

## Supplementary Materials

The following are available online.

## References

[1] REGULATION (EC) No 1907/2006 OF THE EUROPEAN PARLIAMENT AND OF THE COUNCIL of 18 December 2006 concerning the Registration, Evaluation, Authorisation and Restriction of Chemicals (REACH), establishing a European Chemicals Agency, amending Directive 1999/45/EC and repealing Council Regulation (EEC) No. 793/93 and Commission Regulation (EC) No. 1488/94 as well as Council Directive 76/769/EEC and Commission Directives 91/155/EEC, 93/67/EEC, 93/105/EC and 2000/21/EC. https://eur-lex.europa.eu/legal-content/EN/TXT/?uri=CELEX%3A02006R1907-20140410.

[2] Drohmann D., Townsend M. (2013). REACH Best Practice Guide to Regulation (EC) No 1907/2006.

[3] Cozigou G., Crozier J., Hendriksen C., Manou I., Ramirez-Hernandez T., Weissenhorn R. (2015). The European Partnership for Alternative Approaches to Animal Testing (EPAA): Promoting Alternative methods in Europe and Beyond. J. Am. Assoc. Lab. Anim. Sci..

[4] (2017). Guidance on Information Requirements and Chemical Safety Assessment Chapter R.7a: Endpoint Specific Guidance.

[5] Ames B.N., McCann J., Yamasaki E. (1975). Methods for Detecting Carcinogens and Mutagens with the Salmonella/Mammalian-Microsome Mutagenicity Test. Mutat. Res..

[6] Li A.P., Gupta R.S., Heflich R.H., Wasson J.S. (1988). A Review and Analysis of the Chinese Hamster Ovary/Hypoxanthine Guanine Phosphoribosyl Transferase System to Determine the Mutagenicity of Chemical Agents: A Report of Phase III of the U.S. Environmental Protection Agency Gene-tox Program. Mutat. Res..

[7] Parry J.M., Sors A. (1993). The detection and assessment of the aneugenic potential of environmental chemicals: The European Community aneuploidy project. Mutat. Res..

[8] Organization for Economic Cooperation and Development (OECD) (2016). OECD Overview of the Set of OECD Genetic Toxicology Test Guidelines and Updates Performed in 2014–2015.

[9] Gramatica P. (2007). Principles of QSAR models validation: Internal and external. QSAR Comb. Sci..

[10] Creutzenberg O., Koch W., Hansen T., Knebel J., Schuchardt S. (2017). Methodology for the Identification of Granular Biopersitent Particles (GBP) at Workplaces.

[11] Moss O. (1979). Simulants of lung interstitial fluid. Health Phys..

[12] Pelfrêne A., Cave M.R., Wragg J., Douay F. (2017). In Vitro Investigations of Human Bioaccessibility from Reference Materials Using Simulated Lung Fluids. Int. J. Environ. Res. Public. Health.

[13] Ylä-Mononen L. (2015). Decision on a Compliance Check of a Registration Pursuant to Article 41(3) of Regulation (EC) No 1907/2006.

[14] Gebbie P. An Operators Guide for Water Treatment Coagulants. Proceedings of the 31st Annual Qld Water Industry Workshop—Operations Skills, University Central Queensland.

[15] Langelier W.F. (1921). Coagulation of Water With Alum by Prolonged Agitation. Eng. News-Rec..

[16] Akitt J.W., Farnsworth J.A. (1985). Nuclear Magnetic Resonance and Molar Volume Studies of the Complex Formed between Aluminium(III) and the Sulfate Anion. J. Chem. Faraday Trans..

[17] Thompson A.R., Kunwar A.C., Gutowski H.S., Oldfield E. (1987). Oxygen-17 and Aluminium-27 Nuclear Magnetic Resonance Spectroscopic Investigations of Aluminium(III) Hydrolysis Products. J. Chem. Soc. Dalton Trans..

[18] Wang C.Y., Bi S.P., Luo M.B. (2002). Advancement of Studies on the Formation of Polynuclear Hydroxyl Aluminum Species and Their Transformation Laws in Aqueous Systems and Soil Solutions: A Review. ACS Symp. Ser. Am. Chem. Soc..

[19] Johansson G., Lundgren G., Sillén L.G., Söderquist R. (1960). On the Crystal Structure of a basic Aluminium Sulfate and the Corresponding Selenate. Acta Chem. Scand..

[20] Keggin J.F. (1934). The structure and formula of 12-phosphotungstic acid. Proc. R. Soc. Ser. A.

[21] Brosset C. (1952). On the Reactions of the Aluminium Ion with Water. Acta Chem. Scand..

[22] Brosset C., Biedermann G., Sillen L.G. (1954). Studies on the Hydrolysis of Metal Ions. XI. The Aluminium Ion, Al^3+^. Acta Chem. Scand..

[23] Sillen L.G. (1954). On Equilibria in Systems with Polynuclear Complex Formation. I. Methods of Deducing the Composition of the Complexes from Experimental Data. “Core + Links” Complexes. Acta Chem. Scand..

[24] Bi S., Wang C., Cao Q., Zhang C. (2004). Studies on the mechanism of hydrolysis and polymerization of aluminium salts in aquous solutions: Correlations between the “Core-links“ model and “Cage-like” Keggin-Al13 model. Coord. Chem. Rev..

[25] Allouche L., Gerardin C., Loiseau T., Ferey G., Taulelle F. (2000). Al30: A Giant Aluminum Polycation. Angew. Chem. Int. Ed..

[26] Akitt J.W. (1989). Multinuclear Studies of Aluminium Compounds. Prog. Nucl. Magn. Reson. Spectrosc..

[27] Haouas M., Taulelle F., Martineau C. (2016). Recent Advances in Application of ^27^Al NMR Spectroscopy to Materials Science. Prog. Nucl. Magn. Reson. Spectrosc..

[28] Hiradate S. (2004). Speciation of aluminum in soil environments. Soil Sci. Plant Nut..

[29] Casey W.H. (2006). Large Aqueous Aluminum Hydroxide Molecules. Chem. Rew..

[30] Swaddle T.W. (2001). Silicate complexes of aluminum(III) in aqueous systems. Coord. Chem. Rew..

[31] Man P.P., Meyers A. (2000). Quadrupole Couplings in Nuclear Magnetic Resonance, General, in Encyclopedia of Analytical Chemistry.

[32] Vogels R.J.M.J., Kloprogge J.T., Geus J.W. (2005). Homogeneous Forced Hydrolysis of Aluminium Through the Thermal Decomposition of Urea. J. Colloid Interface Sci..

[33] Stryker L.J., Matijevic E. (1969). Counterion complexing and sol stability. II. Coagulation effects of aluminum sulfate in acidic solutions. J. Phys. Chem..

[34] Nishide T., Tsuchaya R. (1965). The formation of Al^3+^-SO_4_^2−^ Ion-pair in an Aqueous Solution of Potassium Aluminium Alum. Bull. Chem. Soc. Jpn..

[35] Hinton J.F., Briggs R.W., Harris R.K., Mann B.E. (1978). Group III-Aluminium, Galium, Indium and Thallium In NMR and the Periodic Table.

[36] Zolnai Z., Juranic N., Vikic-Topic D., Macura S. (2000). Quantitative Determination of Magnetization Exchange Rate Constants from a Series of Two-Dimensional Exchange NMR Spectra. J. Chem. Inf. Comput. Sci..

[37] Lu J., Ma D., Hu J., Tang W., Zhu D. (1988). Nuclear Magnetic Resonance Spectroscopic Studies of pyridine methyl derivatives binding to cytochrome c. J. Chem. Soc. Dalton Trans..

[38] Smith M.E., van Eck E.R.H. (1999). Recent advances in experimental solid state NMR methodology for half-integer spin quadrupolar nuclei. Prog. Nucl. Magn. Reson. Spectrosc..

[39] Man P.P., Couty R., Fraissard J. (1990). Determination of line intensities of ^27^Al in A12O3 by Solid-State NMR. J. Magn. Res..

[40] Alemany L.B., Callender R.L., Barron A.R., Steuernagel S., Iuga D., Kentgens A.P.M. (2000). Single-Pulse MAS, Selective Hahn Echo MAS, and 3QMAS NMR Studies of the Mineral Zoisite at 400, 500, 600, and 800 MHz. Exploring the Limits of Al NMR Detectability. J. Phys. Chem. B.

[41] Fyfe C.A., Bretherton J.L., Lam L.Y. (2001). Solid-State NMR Detection, Characterization, and Quantification of the Multiple Aluminum Environments in US-Y Catalysts by 27Al MAS and MQMAS Experiments at Very High Field. J. Am. Chem. Soc..

[42] Grey C.P., Vega A.J. (1995). Determination of the Quadrupole Coupling Constant of the Invisible Aluminum Spins in Zeolite HY with ^1^H/^27^Al TRAPDOR NMR. J. Am. Chem. Soc..

[43] Shafran K.L., Perry C.C. (2005). A systematic investigation of aluminium ion speciation at high temperature. Part. 1. Solution studies. Daltom Trans..

[44] Zhao H., Liu H., Qu J. (2009). Effect of pH on the aluminum salts hydrolysis during coagulation process: Formation and decomposition of polymeric aluminum species. J. Colloid Interf. Sci..

[45] Sarpola A., Hietapelto V., Jalonen J., Jokela J., Laitinen R.S. (2004). Identification of the hydrolysis products of AlCl_3_·6H_2_O by electrospray ionization mass spectrometry. J. Mass. Spectrom..

[46] Akitt J.W., Farthing A. (1978). New ^27^Al Studies of the Hydrolysis of the Aluminium(III) Cation. J. Magn. Reson..

[47] Majorek K.A., Porebski P.J., Dayal A., Zimmermann M., Jablonska K., Stewart A.J., Chruszcz M., Minor W. (2012). Structural and immunologic characterization of bovine, horse, and rabbit serum albumins. Mol. Immunol..

[48] Bal W., Sokolowska M., Kurowska E., Faller P. (2013). Binding of transition metal ions to albumin: Sites, affinities and rates. Biochim. Biophys. Acta.

[49] Fatemi S.J.A., Kadir F.H.A., Moore J.A. (1991). Aluminium transport in blood serum, Binding of aluminium by human transferrin in the presence of human albumin and citrate. Biochem. J..

