# Supplementary material for: 27Al NMR Study of the pH Dependent Hydrolysis Products of Al2(SO4)3 in Different Physiological Media
Source: Molecules. 2018 Apr 2;23(4):808. doi: 10.3390/molecules23040808 (PMC6017892; doi:10.3390/molecules23040808)
Supplement: Supplementary file 1 [file molecules-23-00808-s001.pdf]

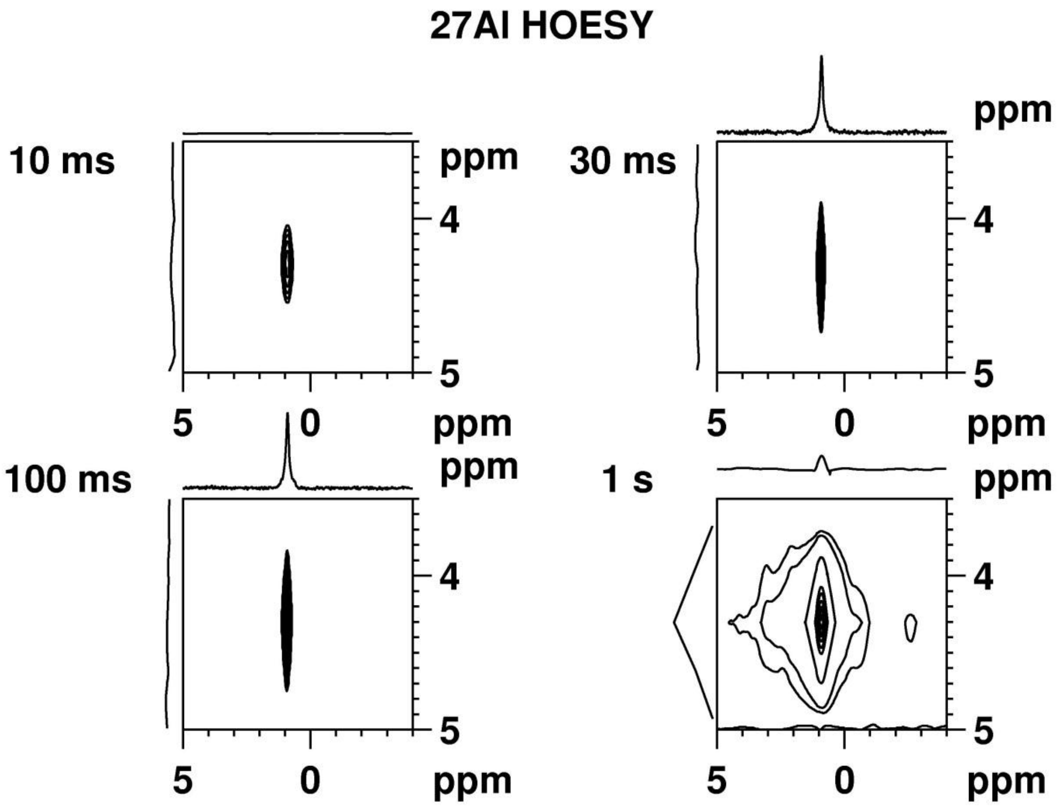

Figure S1. ^{27}Al HOESY NMR spectra of 50 g/L $Al_2(SO_4)_3$ dissolved in media L1 recorded with resp. 10 ms, 30 ms, 100 ms and 1 s mixing times.

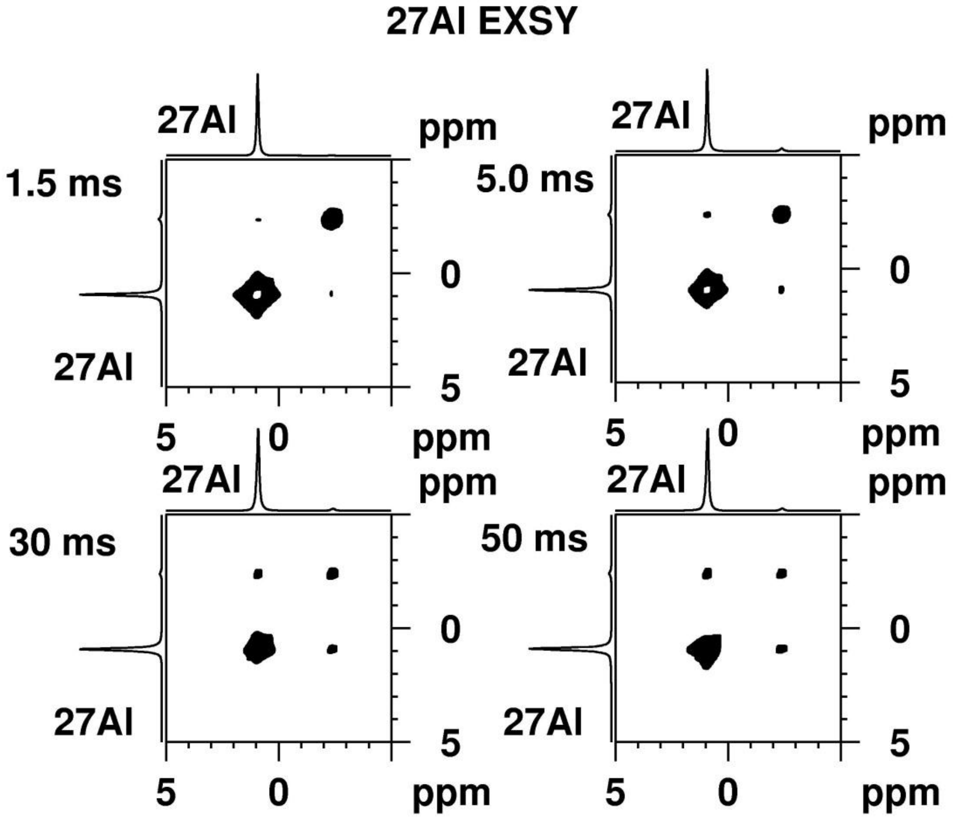

Figure S2. ^{27}Al EXSY NMR spectra of 50 g/L $Al_2(SO_4)_3$ dissolved in media L1 recorded with 1.5 ms (up left), 5.0 ms (up right), 30 ms (bottom left) and 50 ms (bottom right) mixing times. The evolution of the diagonal and cross-peaks intensity is presented as a function of the mixing time.

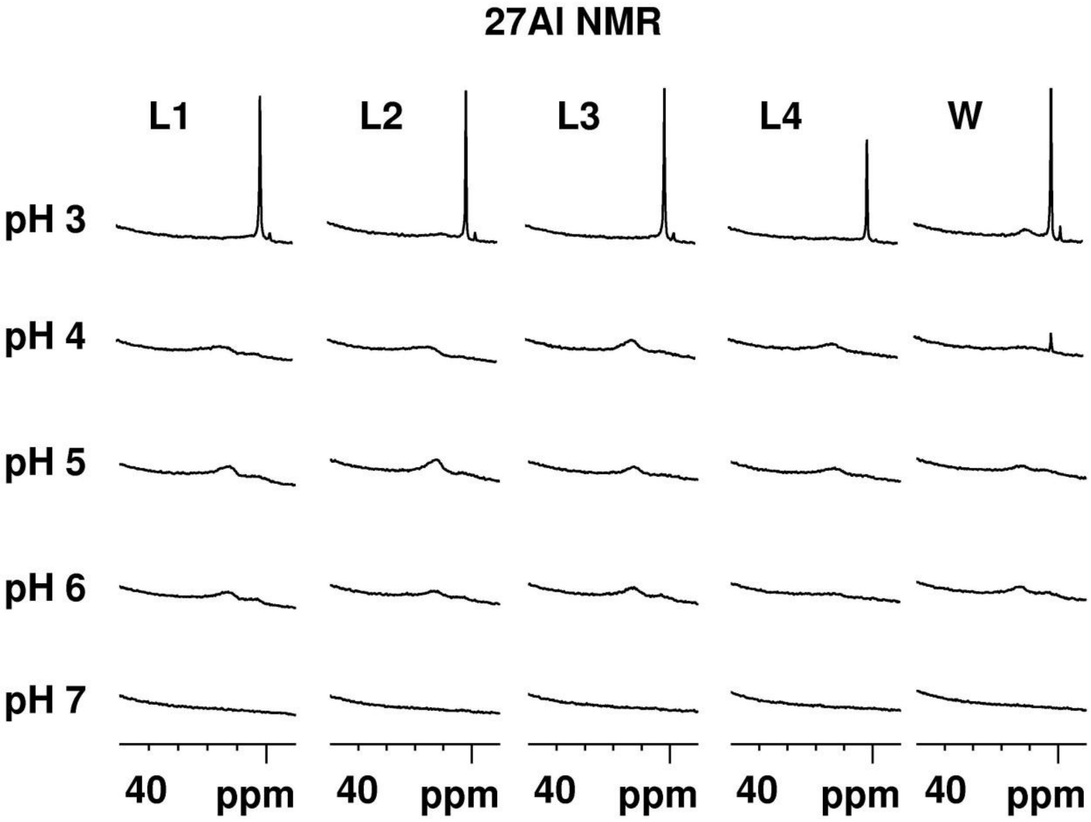

Figure S3. ^{27}Al NMR spectra of $Al_2(SO_4)_3$ with a concentration of 5 g/L dissolved in the media L1-4 and water as a function of pH.

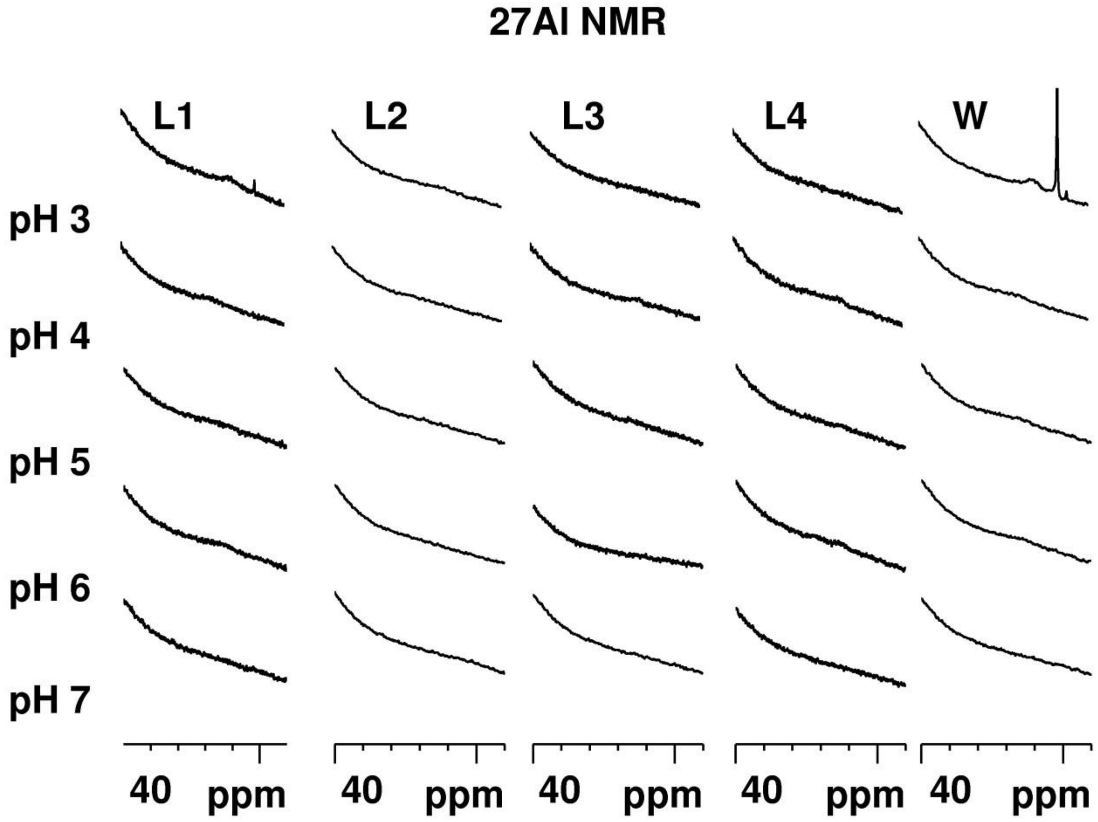

Figure S4. ^{27}Al NMR spectra of $Al_2(SO_4)_3$ with a concentration of 0.5 g/L dissolved in the media L1-4 and water as a function of pH. The spectra were measured with 1024 scans. In all spectra excluding the ones at pH 3 for the water and L1 solutions aluminium signal was barely detected.

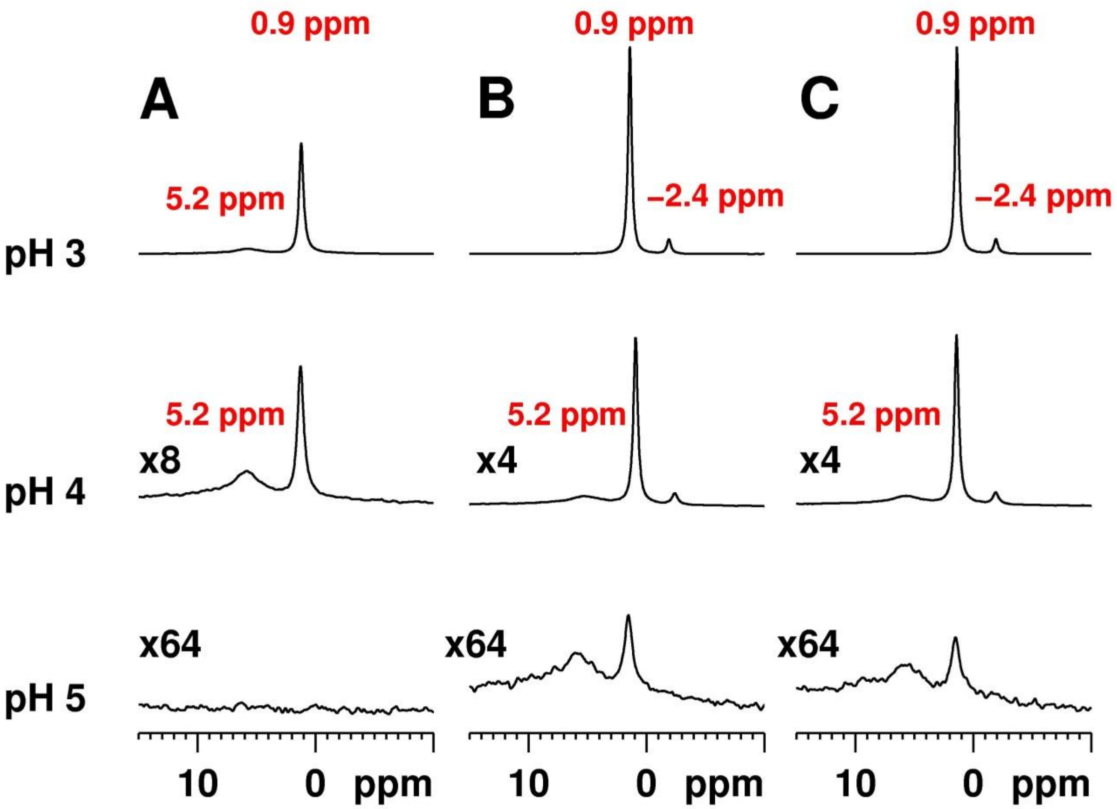

Figure S5. ^{27}Al NMR spectra of $AlCl_3$ in H_2O with a concentration of 50 g/L (A), $Al_2(SO_4)_3$ in H_2O with a concentration of 50 g/L (B) and $Al_2(SO_4)_3$ in H_2O + 1% penicillin with a concentration of 50 g/L (C) at pH 3, 4 and 5. The background was measured in an additional experiment and subsequently subtracted from all spectra. No deuterium lock was used. The signal of the hexaaquo-complex $[Al(H_2O)_6]^3$ was detected in all spectra except the one of the $AlCl_3$ solution at pH 5. The resonances for the were detected in the spectra of aluminium sulfate at pH 3 and 4. Such were not observed in the aluminium chloride solutions independent of the concentration. The presence of penicillin resulted in a slight reduction of the intensity of the monomer signal at pH 5. Clearly the type of salt/counter ion at the same concentration and pH has an effect on the hydrolysis products formed.

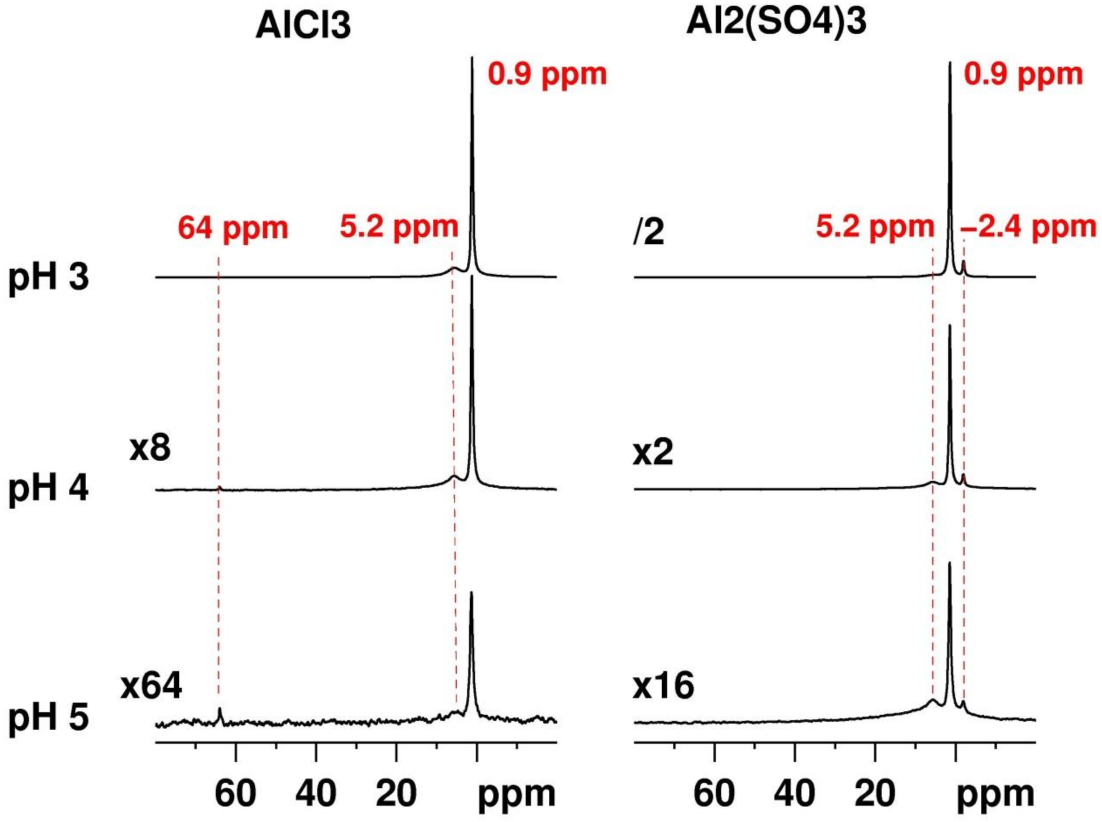

Figure S6. ^{27}Al NMR spectra of saturated aqueous solutions of $AlCl_3$ (left) and $Al_2(SO_4)_3$ (right) at pH 3, 4 and 5. The background was measured in an additional experiment and subsequently subtracted from all spectra. No deuterium lock was used. The signals of the hexaaquo-complex $[Al(H_2O)_6]^3$ and the dimer $[Al_2(OH)_2(H_2O)_8]^{4+}$ were observed in all presented spectra. No resonance for the $[Al(H_2O)_5SO_4]^+$ were detected in the saturated solution of $AlCl_3$ independent of the pH. Instead a new resonance at 64.0 ppm for the central tetra-coordinated aluminium AlO_4 in $AlO_4Al_{12}(OH)_{24}(H_2O)_{12}{}^{7+}$ (A13) appeared at pH 4 and 5. The type of salt/counter ion at the same concentration and pH has an effect on the hydrolysis products formed.

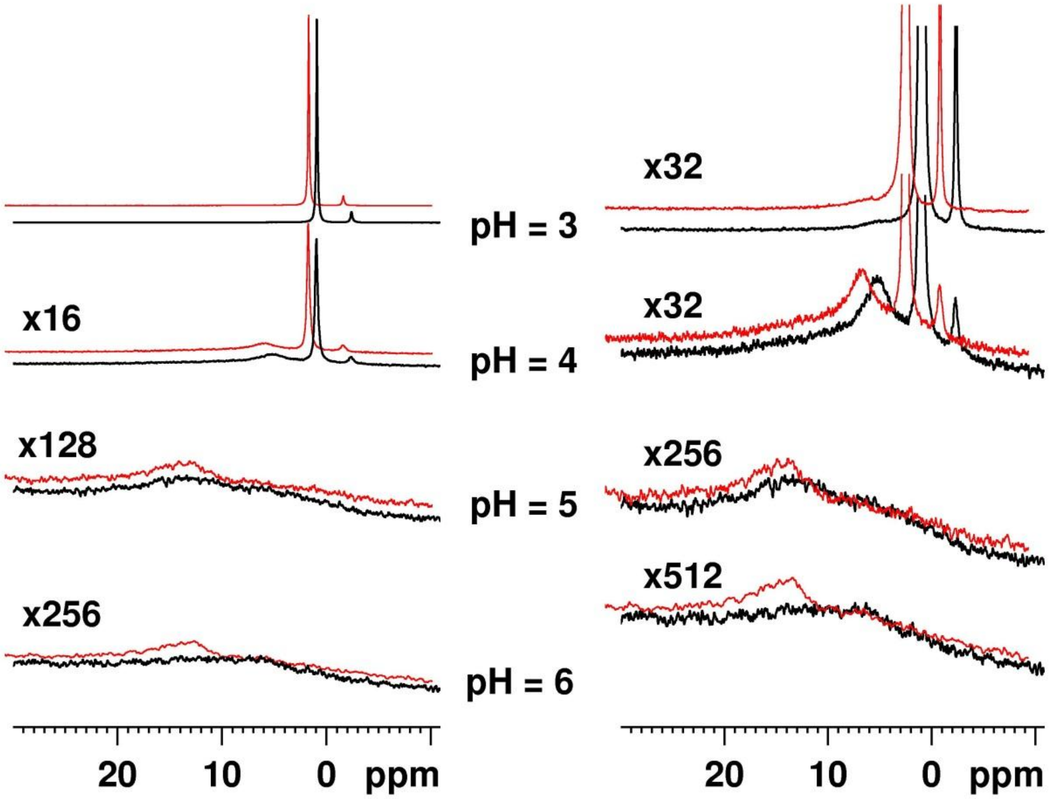

Figure S7. ^{27}Al NMR spectra of $Al_2(SO_4)_3$ recorded in L1 medium at pH 3 to 6 as prepared (black) and 6 weeks after storing at ambient conditions (red).
